# Making CT Dose Monitoring Meaningful: Augmenting Dose with Imaging Quality

**DOI:** 10.3390/tomography9020065

**Published:** 2023-04-07

**Authors:** Njood Alsaihati, Francesco Ria, Justin Solomon, Aiping Ding, Donald Frush, Ehsan Samei

**Affiliations:** 1 Carl E. Ravin Advanced Imaging Laboratories, Department of Radiology, Duke University Medical Center, Durham, NC 27710, USA; francesco.ria@duke.edu (F.R.); justin.solomon@duke.edu (J.S.); aiping.ding@duke.edu (A.D.); donald.frush@duke.edu (D.F.); esi.samei@duke.edu (E.S.); 2 Clinical Imaging Physics Group, Department of Radiology, Duke University Medical Center, Durham, NC 27710, USA; 3 Center for Virtual Imaging Trials, Department of Radiology, Duke University Medical Center, Durham, NC 27710, USA

**Keywords:** CT, dose monitoring, radiation dose, image quality, quality assurance

## Abstract

Due to the concerns about radiation dose associated with medical imaging, radiation dose monitoring systems (RDMSs) are now utilized by many radiology providers to collect, process, analyze, and manage radiation dose-related information. Currently, most commercially available RDMSs focus only on radiation dose information and do not track any metrics related to image quality. However, to enable comprehensive patient-based imaging optimization, it is equally important to monitor image quality as well. This article describes how RDMS design can be extended beyond radiation dose to simultaneously monitor image quality. A newly designed interface was evaluated by different groups of radiology professionals (radiologists, technologists, and physicists) on a Likert scale. The results show that the new design is effective in assessing both image quality and safety in clinical practices, with an overall average score of 7.8 out of 10.0 and scores ranging from 5.5 to 10.0. Radiologists rated the interface highest at 8.4 out of 10.0, followed by technologists at 7.6 out of 10.0, and medical physicists at 7.5 out of 10.0. This work demonstrates how the assessment of the radiation dose can be performed in conjunction with the image quality using customizable user interfaces based on the clinical needs associated with different radiology professions.

## 1. Introduction

In recent years, concerns regarding radiation dose and its potential risk to patients undergoing diagnostic examinations have been raised by the scientific community and regulatory organizations [1]. In particular, new requirements have been brought forth regarding radiation burden in medical imaging [2,3,4]. Radiation dose monitoring systems (RDMSs), especially for computed tomography (CT), have been implemented in medical imaging departments aiming towards regulatory compliance while continuing to provide valuable information concerning the optimization and justification of radiological procedures [5].

Because patient radiation exposure has been the primary focus of regulatory requirements, currently available RDMSs are mostly focused on radiation burden-related information only. However, as stated recently in the International Commission on Radiological Protection (ICRP) publication 135, the radiological procedure’s diagnostic information should be assessed in terms of the quality of the images that are essential for the diagnostic task [6]. Therefore, a comprehensive evaluation of the performance of a radiological procedure cannot be based on radiation dose alone, and the provided quality of the diagnostic information should also be evaluated simultaneously. This augmentation of the examination dose metrics in the monitoring paradigm can then better inform the comprehensive patient-based value by addressing the radiation’s safety and the consistent diagnostic benefit, which is currently not available with existing RDMSs.

The purpose of the following material is to describe how the RDMS’s design can be extended to assess the radiation dose and image quality simultaneously. In particular, by adapting effective and efficient data visualization, a new RDMS interface was designed. Moreover, the system interface was evaluated by radiology professionals, consisting of radiologists, technologists, and medical physicists, in terms of its effectiveness at providing relevant insights that incorporate both dose and quality in clinical practice.

## 2. Materials and Methods

Integral to any RDMS is the nature and organization of the imaging data that need to be thoroughly examined in order to design and implement an effective and robust monitoring system. The section, thus, first focuses on the nature of the data associated with CT practices. Following this, the metrology of extracting meaningful quantities to monitor is described with particular focus on radiation dose metrics, image quality metrics, patient information, scanner parameters, and operational factors. Finally, this section introduces the proposed RDMS design and interface, which is further evaluated for clinical usefulness by radiology professionals.

### 2.1. The Nature of the Data

Clinical CT data generally contain highly heterogeneous metadata due to a lack of consensus on procedure naming standards, a lack of effective centralized protocol management systems, and the complex realities of providing radiological services. It is not uncommon for a large healthcare provider to offer hundreds of different CT examination labels. These heterogeneous metadata make it difficult to properly aggregate like-examinations for large-scale retrospective statistical analysis and are also problematic for real-time alerting features. Both inter and intra provider heterogeneities are problematic. An effective RDMS needs to implement a robust method for homogenizing the data. Such metadata include:

- Labels and attributes: study description, protocol name, anatomical region, scanner model, etc.
- Patient data: sex, age, body mass index (BMI), effective diameter, water-equivalent diameter (WED), etc.

### 2.2. Meaningful Quantities to Monitor

A comprehensive RDMS should monitor not only radiation dose information but all CT exam-related data, which can be categorized into image quality attributes, patient information, scanner parameters, and operational factors [7]. Within each monitored data category, the relevant quantities to monitor are described below.

#### 2.2.1. Dose Metrics

Various metrics of radiation exposure have been used over the years by the radiology community (Table 1). Each metric provides a different radiation burden depiction and has its own limitations. Therefore, careful usage and evaluation should be considered to inform clinical practice and the accurate justification and optimization of CT procedures [8]. For example, the volume computed tomography dose index (CTDI_vol_) and dose length product (DLP) both represent the radiation output of a CT scanner, but they do not account for any patient attributes. Therefore, these quantities are effective in comparing between systems and institutions or to establish diagnostic reference levels (DRL) [6], but they should not be used as a patient risk surrogate. As a step forward, the Size-specific dose estimate (SSDE) was developed to overcome this limitation by accounting for some attributes of a patient’s size, but the SSDE still does not account for body shape, organ location, and tissue composition [9]. Furthermore, the effective dose (E) can be used to characterize radiation detriment across a clinical population but cannot represent individual patient risk [10]. Currently, the combination of organ doses (OD) and age- and sex-specific organ sensitivity conversion factors, namely the risk index (RI), can provide the closest surrogate for patient risk [8,11].

#### 2.2.2. Image Quality Metrics

Automated algorithms can be deployed into RDMSs to quantify the relevant image quality metrics from clinical images. For instance, several methods have been developed over the past few years to calculate image quality metrics in vivo, such as noise magnitude, noise texture, and the detectability index (d’) [17,18,19]. With respect to these metrics, it is important to recognize that different reconstruction settings can impact image quality without altering the scanner dose output. Moreover, the evaluation of image quality needs to be considered in the context of the specific clinical task. The detectability index, in particular, relies on noise magnitude, the noise power spectrum, and the resolution combined with a specific task function. Therefore, the evaluation of task-based image quality is also needed in RDMSs to comprehensively assess the performance of radiological procedures in the clinical domain. In this study, the proposed RDMS design focuses on augmenting the radiation dose information with quantitative image quality data in terms of noise magnitude. To achieve this, the system deployed an enhanced version of an earlier algorithm, automatically calculating the global noise index (GNI) in patient images [17]. To estimate the GNI, a [–300, 300] HU threshold was first applied to the CT images to isolate soft tissue. Then, for each slice, a 30 pixels × 30 pixels region of interest was identified around each pixel, generating a standard deviation. The standard deviation maps across the slice were then used to generate a histogram, with the mode of the histogram representing the global noise level of the slice. Finally, the GNI was computed, averaging the global noise levels across the whole study. The algorithm has been validated by conducting a phantom study and an observer study, which indicated that the average absolute difference between the measured global noise index and the phantom image noise is 3.4%, while the difference between the GNI and the observer noise measurement is 4.7% [17].

#### 2.2.3. Patient Information, Scanner Parameters, and Operational Factors

While the dose and quality metrics are the primary factors for monitoring in an RDMS, these metrics are directly affected by a series of other factors. First among them are the category patient factors. Patient demographic data, such as age, sex, BMI, WED, etc., are essential ingredients of an RDMS. They are often used to identify sub-groups of patients, analyze the data accordingly, and in the metrology science, they can be used to derive certain dose metrics (e.g., SSDE, OD).

In addition to patient information, scanner parameters are also crucial in any CT performance monitoring system since the radiation dose and image quality are both outcomes of such settings. It is necessary to understand the impact of quantities, such as X-ray tube voltage (kV), slice thickness, and scan mode, on radiation dose and image quality in the optimization strategies for CT procedures.

Lastly, operational factors inform CT performance. They include details associated with the ordered and performed protocols, and the information linking to the radiologists and technologists responsible for the examination. Such information is essential to evaluate daily clinic workflow and imaging professional workload. These operational factors are relevant as they affect not only clinical consistency but also the individual procedure’s outcome. They can also identify opportunities for targeted training to improve practice.

### 2.3. RDMS Design and Evaluation

#### 2.3.1. RDMS Design

**Figure 1:**
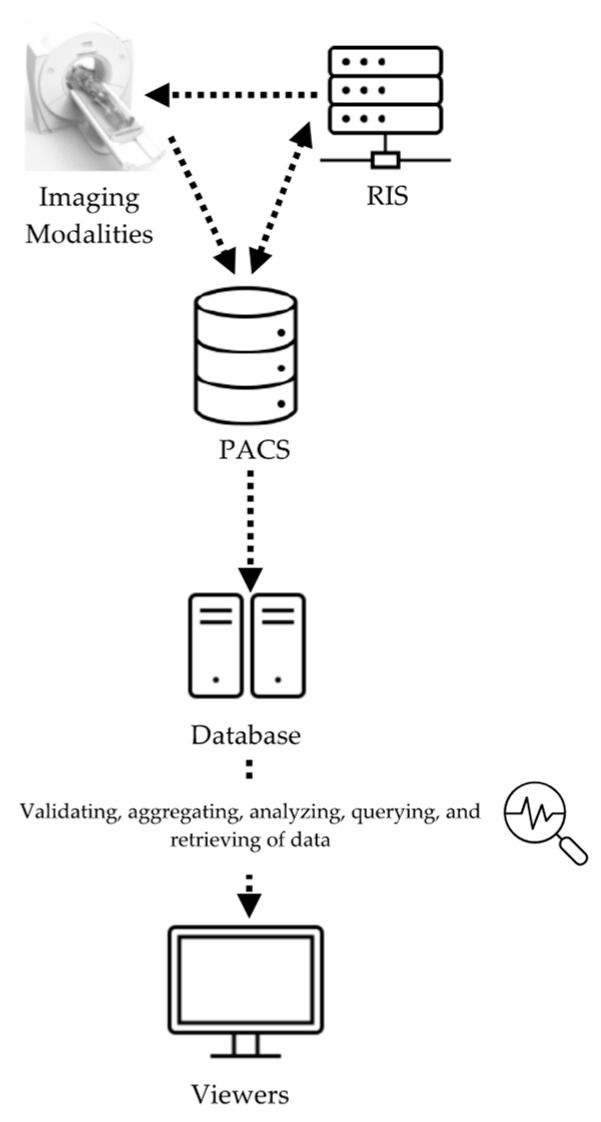
Schema of METIS design.

Considering the desired functionalities and metrics for the RDMS described above, a comprehensive RDMS was developed and implemented at Duke University Medical Center. The system, so-called METrology for Imaging Systems (METIS), was designed to simultaneously assess and record measures of radiation dose and image quality, in conjunction with patient data, scanner parameters, and operational factors. The METIS data collection has three layers: (1) data source layer; (2) intermediary administration layer; and (3) presentation layer (Figure 1). The first layer is a database containing all of the image data and patient information. Data from the radiology information system (RIS) and individual imaging systems are collected through the picture archiving and communication system (PACS). This layer provides multiple application program interfaces (APIs) to connect with the database to enable data insertion, update, and deletion.

Following the first layer, the intermediary administration layer consists of image data access and management, business logic, such as the system’s integration with the PACS, connection with individual imaging systems, validation of data, the aggregation and analysis of data, querying and retrieving data, and data updates. Lastly, the third layer provides a graphical user interface (GUI) where users can view the examination data and input additional information [7].

#### 2.3.2. RDMS Interface

An effective RDMS depends on the useful visualization of the associated data. Towards that goal, we undertook a systematic exploration of key questions that a RDMS should answer, aiming for the transformation of data into knowledge. We then envisioned individual charts, each of which would answer one of these questions. In particular, through showing trends in the dose data and CT systems, variations within patient cohorts, outliers in dose and image quality data (e.g., under/over-exposure cases, high and very low noise images), comparison across systems and institutions, and inconsistencies across systems, operations, and patients [6,7,15,20,21]. From this exercise, 15 charts were deemed most effective to depict an aggregate representation of the information to provide meaningful knowledge to inform quality improvement. The data were then analyzed and aggregated to form these charts. Table 2 reports the description of the charts and the targeted questions.

The interface was then evaluated for clinical usefulness through an observer study. The 15 charts were populated with data from 25,322 clinical chest, abdomen, and pelvic CT examinations from 5925 patients. The data represented diversity in the CT makes and models (GE Healthcare, Waukesha, WI; and Siemens Healthineers, Erlangen, Germany). The observer study deployed 12 radiology professionals who were currently using RDMSs in their institutions and were familiar with the needs and potential utility of the system. The participants included four radiologists, four technologists, and four medical physicists. They evaluated the visualized data in terms of their potential usefulness in assessing the quality and dose-related safety of clinical practices. The charts were scored, using a Likert scale, from very useful = 10, to useful = 8, moderately useful = 6, slightly useful = 4, and not useful = 0.

## 3. Results

Table 3 provides the results of the evaluation for each chart by the 12 radiology professionals divided by sub-groups, as well as the average scores. Table 4 summarizes the overall results of the visualization charts. The overall average score of the 15 charts (Figures S1–S14) was 7.8/10.0, where the radiologists gave the highest average score of 8.4. Chart 10 (Figure 2) received the highest average score of 8.7 while Charts 6 and 9 (Figure 3 and Figure 4) received the lowest average score of 6.2 among the participants. In terms of the difference between the sub-groups of professionals, Charts 2 and 11a (Figure 5 and Figure 6) were scored similarly across the professionals, resulting in a minimum difference of the average score of 0.5. The maximum difference of the average score was 4.5, where the radiologists and technologists rated Chart 4 (Figure 7) with an average score of 10 and 5.5, respectively.

## 4. Discussion

RDMSs have seen increased use in recent years as a means to collect, process, manage, and analyze radiation dose-related information. Such systems can serve as a tool to inform the optimization of radiological procedures [22]. However, given the fact that the need for any radiological exam is based on clinical objectives, such an optimization required the incorporation of both the radiation dose and image quality. In particular, the in vivo image quality metrics can most directly reflect the actual (as opposed to presumed) clinical quality. In this article, we demonstrated a new design of RDMS that provides the simultaneous visualization of the radiation dose and the image quality in terms of noise alone. We further showed the results of its evaluation by radiology professionals. They indicated that the designed charts are useful in facilitating the assessment of the quality and safety of clinical practices, with an overall average score among the radiology professionals of 7.8 out of 10. The charts used different analytical techniques with the purpose of extracting meaningful knowledge related to the overall radiological performance.

The results of our study showed that different charts were favored differently by the participants; different radiology professionals preferred different visualization of the data aggregates. This is expected, as different professions in the radiology department have different roles and responsibilities. Thus, the RDMS dashboard should be customized accordingly based on their needs and responsibilities to facilitate best practice. For instance, medical physicists are primarily focused on the implementation and management of the RDMS, in addition to assuring the high-quality, safety, consistency, and optimum operation of imaging systems. Radiologists are interested in the overall radiation dose and image quality to ensure the patient’s safety and effective diagnosis. Technologists are responsible for acquiring high-quality images towards an accurate and consistent imaging outcome and do so while assuring patient safety. For instance, when comparing radiation exposure with DRL and AD, technologists seem to prefer a visualization that reports the overall institution’s performance (Figure S14) rather than visualizations that focus on specific data sets (Figures S4 and S5). In addition, administrators would be interested in the information provided by the RDMS, to evaluate the overall institution’s performance and safety. Ultimately, all visualizations should inform consistent practice through a team effort, where outliers or technical problems are identified, and protocols reviewed and adjusted towards continuous improvement in patient care and efficient workflow.

In recent years, a great number of commercial RDMSs have become available and are used worldwide [22,23]. These systems have different data monitoring levels and approaches. A common approach considers only radiation dose monitoring together with patient data, scanner parameters, and study information. A second approach also includes qualitative image quality information in terms of user preference. A third approach incorporates quantitative image quality information. All the approaches can be useful for understanding, managing, and mitigating radiation dose concerns together with operation evaluation and consistency. However, the first two approaches do not consider the quantitative and objective assessment of image quality, which is essential in the comprehensive evaluation of a radiological procedure. In this article, the proposed system follows the third approach, which aligns with the ICRP and IAEA recommendations to inform patient-based optimization, completely embodying the essence of the ALARA principle: as low as reasonably achievable while the acquisition of acceptable clinical images is ensured [6,24].

**Figure 8:**
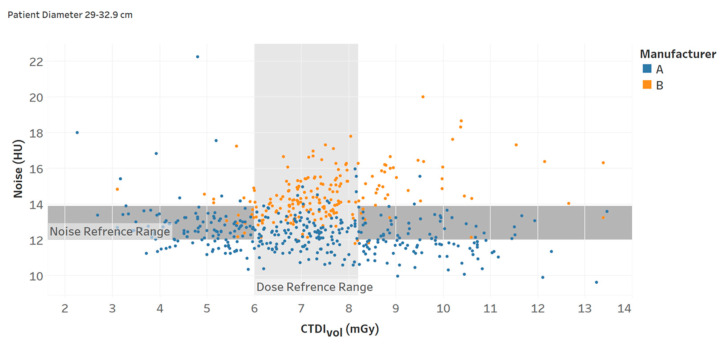
Noise and CTDI_vol_ reference ranges (interquartile intervals) for a specific protocol (Chart 13).

Recent studies have demonstrated that augmenting the radiation dose information with quantitative image quality data can facilitate a more comprehensive and efficient assessment of CT practice. Specifically, two recent studies have shown that establishing the reference levels for both the radiation dose and image noise can improve the evaluation of CT imaging procedures [21,25]. Such implementation can help identify very low-quality and high dose cases. Figure 8 illustrates an example of this. Additionally, analyzing the patient population data using both reference levels can provide essential information to optimize CT imaging protocols, taking into consideration factors that influence such optimization across the diversity of patients in clinical practice; for example, considering the influence of automated tube current modulation (ATCM) when such technology is used.

This study had a few limitations. The observer study was based on a limited sample of imaging professionals. However, the observer study participants were selected based on their familiarity and knowledge of the needs and potential utility of the RDMS system and were reasonable representatives of radiologists, technologists, and medical physicists. Future investigations can explore the Radiation Dose Monitoring System’s potential for other radiology professionals, particularly department administrators. In addition, 15 charts were proposed because they provide a descriptive sample of the METIS analytical features. Future studies can provide a multiplicity of graphical data aggregations that can represent different clinical scenarios, designed towards the extraction of actionable knowledge and the retrospective evaluations that can inform decision-making. The study considered specific radiation dose metrics, such as CTDI_vol_ and E_OD_, and image quality in terms of noise only. Future extensions will consider other radiation dose and image quality metrics, such as SSDE, risk index, image resolution, and d’, as well as the effect of image artifacts. Lastly, the study focused on the RDMS’s implementation in computed tomography only; however, the current system can be extended to include other imaging modalities (namely mammography, fluoroscopy, radiography, and nuclear imaging).

## 5. Conclusions

The RDMS has become an essential tool in imaging departments to inform the optimization and justification of radiological procedures. To enable a more comprehensive performance assessment and the transformation of data into knowledge, the RDMS should be extended beyond dose monitoring to simultaneously include image quality. In addition, the RDMS should provide customized data analysis and visualization for different radiology professionals based on their associated clinical priorities.

## Figures and Tables

**Figure 2:**
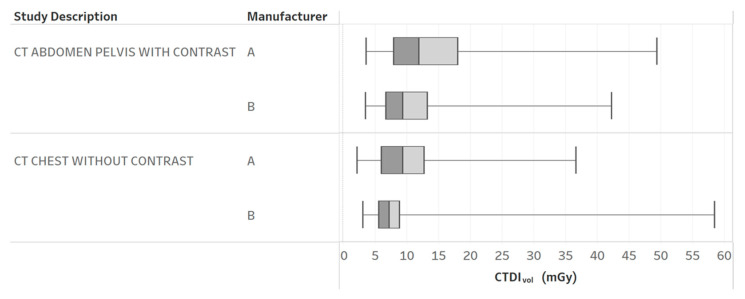
CTDI_vol_ boxplots by different CT protocols and manufacturers. Chart 10: Most highly ranked.

**Figure 3:**
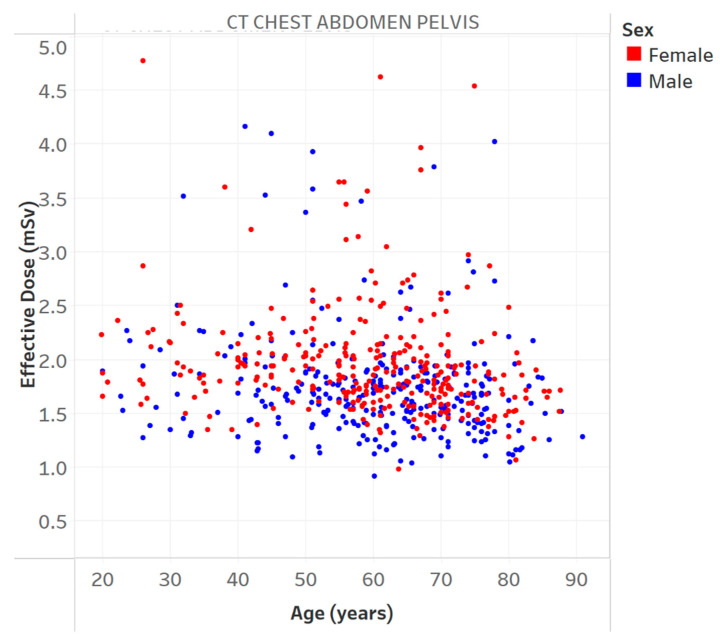
Effective dose vs. age for a specific protocol. Chart 6: Least highly ranked.

**Figure 4:**
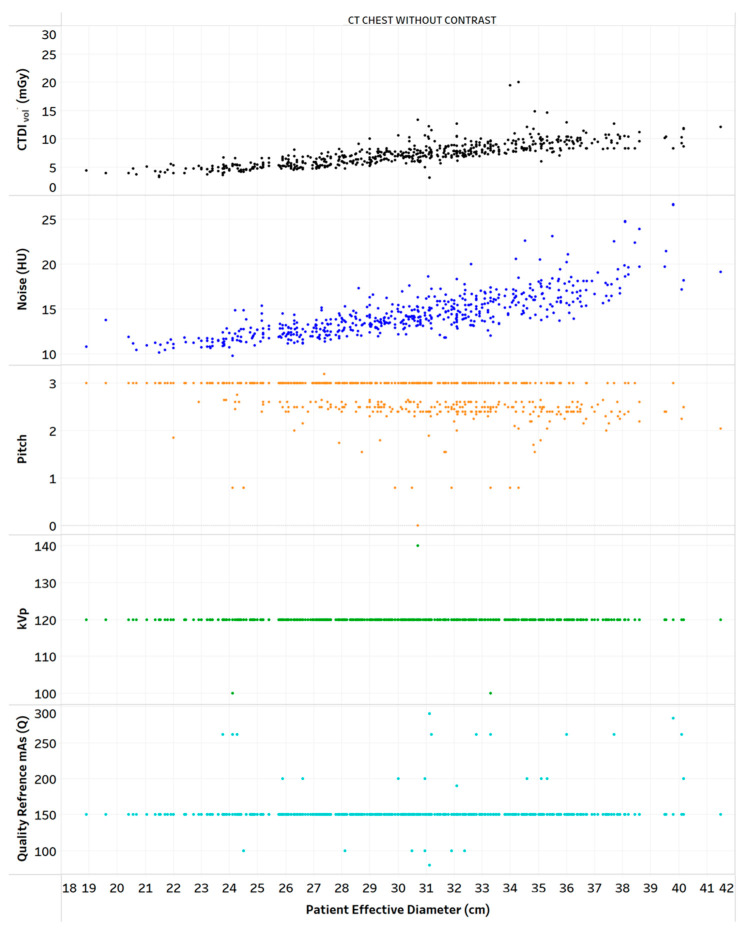
Distribution of CTDI_vol_, noise, and scanning parameters vs. patient diameter for one protocol. Chart 9: Least highly ranked.

**Figure 5:**
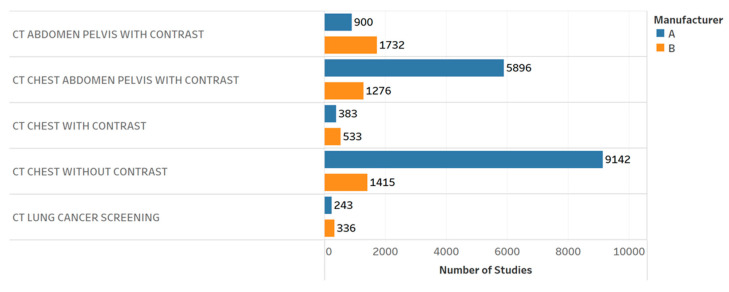
Distribution of CT exams by different protocols and manufacturers. Chart 2: Scored similarly across the professionals.

**Figure 6:**
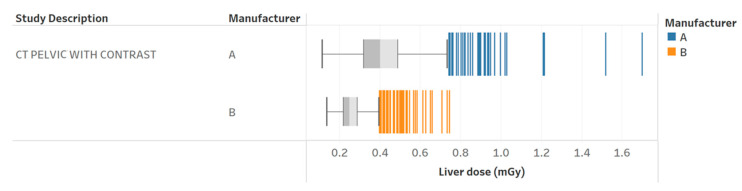
Radiation dose to the liver for pelvic with contrast exams for two different scanners. Chart 11a: Scored similarly across the professionals.

**Figure 7:**
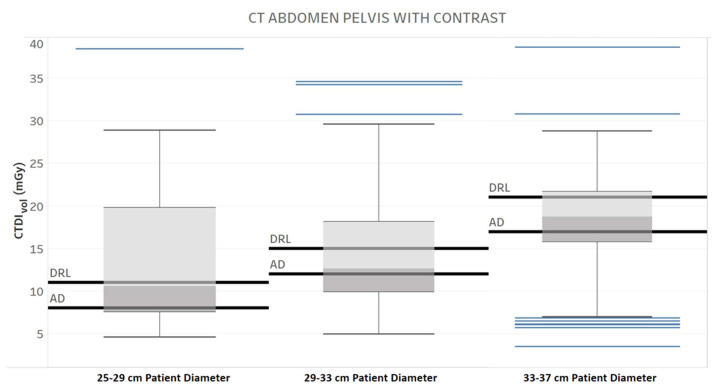
Diagnostic Reference Level (DRL) and Achievable Dose (AD) for three different patient diameter ranges, one protocol, and one manufacturer. Chart 4: The maximum score difference between the professionals.

**Table 1:** List of radiation dose surrogates.

Index	Definition	Representing
CTDI_vol_	Volume computed tomography dose index	Standardized measure of radiation output of a CT scanner in a specified phantom [12].
DLP	Dose length product	Radiation output of a CT scanner over the length of a scan [12].
SSDE	Size-specific dose estimate	Radiation output of a CT scanner that takes a patient’s attributes of size into account [13].
OD	Organ absorbed dose(s)	Estimated dose absorbed to a specific organ in the body [14].
E_OD_	Effective dose calculated from the estimated organ doses	Effective dose calculated based on the estimated organ doses of the patient, incorporating organ sensitivities [15].
RI	Risk index	Radiation risk index calculated based on the estimated organ doses of the patient, incorporating weighted sensitivities based on organ, age, and sex [16].

**Table 2:** Description of the charts and the associated key questions that a RDMS should answer.

Chart	Description	Question Targeted
1	CTDI_vol_ for patient effective diameter of all CT exams.	Is the administered radiation dose reflective of patient size?
2	Distribution of CT exams by different protocols and manufacturers.	What is the scanners’ workload?
3	Distribution of the CTDI_vol_ by different protocols, manufacturers, and patient effective diameter.	Is the radiation burden related to different protocols, scanners, and patient size?
4	Diagnostic reference level (DRL) and achievable dose (AD) for three different patient diameter ranges for one protocol and manufacturer.	How is the radiation output of the scanner compared with the literature and regulatory standards across patient body habitus? [20]
5	DRL and AD for one protocol and two manufacturers.	How is the radiation output of the scanner compared with the literature and regulatory standards across different scanners? [20]
6	Effective dose vs. age for a specific protocol.	Is the effective dose related to patient age?
7	Distribution of the CTDI_vol_ and effective dose vs. patient diameter for one protocol and one manufacturer.	How are different metrics of the radiation dose related to patient body habitus?
8	Distribution of noise for different slice thicknesses and CTDI_vol_ vs. patient diameter for one manufacturer across two different protocols.	How does the radiation dose and image quality change with patient size in different protocols and reconstructions?
9	Distribution of CTDI_vol_, noise, and scanning parameters vs. patient diameter for one protocol.	How does the scanner’s parameters affect the radiation dose and image quality in the patient population?
10	CTDI_vol_ boxplots by different protocols and manufacturers.	How does the radiation dose distribution change across scanners and protocols?
11a	Radiation dose to the liver for pelvic CT with contrast exams for two different scanners.	How does the dose to a specific organ change across scanners?
11b	Scanning parameter comparison between a specific patient (outlier) and the whole population.	What is the underlying reason behind an outlier?
12	Median organ doses and effective dose for chest without contrast and pelvic with contrast CT exams.	How does the organ radiation dose distribution and effective dose change with different protocols?
13	Noise and CTDI_vol_ reference ranges (interquartile intervals) for a specific protocol.	How are the radiation dose and noise magnitude compared with the literature and regulatory standards for different patient body habitus? [21]
14	Comparison of institution average CTDI_vol_ with AD and DRL for one protocol	How is the radiation dose distribution at the institution compared with the regulatory levels in a patient population? [20]

**Table 3:** Results of the evaluation of each chart by the sub-groups of radiology professionals.

Professionals	Charts														
	1	2	3	4	5	6	7	8	9	10	11a	11b	12	13	14
Radiologists	10	8	9	10	8	6.5	7	8.5	7	9	7	9.5	8.5	9.5	8
Technologists	6	7.5	9	8	7.5	6.5	7.5	8	5.5	9	8	8	7	8.5	7.5
Medical physicists	8.5	7.5	7.5	5.5	6	5.5	9	7.5	6	8	7.5	8	10	7	9
All	8.2	7.7	8.5	7.8	7.2	6.2	7.8	8	6.2	8.7	7.5	8.5	8.5	8.3	8.2

**Table 4:** Summary of the overall results of the visualization charts by the sub-groups of radiology professionals.

Professionals	Average Score (out of 10)	Range	Variability
Radiologists	8.4	6.5–10	1.14
Technologists	7.6	5.5–10	1.34
Medical physicists	7.5	5.5–9	1.00
All	7.8	5.5–10	0.79

## Data Availability

Data is available upon request from the corresponding author.

## Supplementary Materials

The following supporting information can be downloaded at: https://www.mdpi.com/article/10.3390/tomography9020065/s1, Figure S1: CTDI_vol_ for patient effective diameter of all CT exams. Figure S2: Distribution of CT exams by different protocols and manufacturers. Figure S3: Distribution of the CTDI_vol_ by different protocols, manufacturers, and patient effective diameter. Figure S4: Diagnostic reference level (DRL) and achievable dose (AD) for three different patient diameter ranges, one protocol, and one manufacturer. Figure S5: Diagnostic reference level (DRL) and achievable dose (AD) for one protocol and two manufacturers. Figure S6: Effective Dose vs age for a specific protocol. Figure S7: Distribution of the CTDI_vol_ and effective dose vs patient diameter for one protocol and one manufacturer. Figure S8: Distribution of noise and CTDI_vol_ vs patient diameter for one manufacturer across two different protocols. Images were reconstructed with two different slice thicknesses. Figure S9: Distribution of CTDI_vol_, noise, and scanning parameters vs patient diameter for one protocol. Figure S10: CTDIvol boxplots by different protocols and manufacturers. Figure S11a: Radiation dose to the liver for pelvic with contrast exams for two different scanners. Figure S11b:Scanning parameters comparison between a specific patient (outlier) and the whole population. Figure S12: Median organ doses and effective dose for chest without contrast and pelvic with contrast exams. Figure S13: Noise and CTDI_vol_ reference ranges (interquartile intervals) for a specific protocol. Figure S14: Comparison of institution average CTDI_vol_ with achievable dose (AD) and diagnostic reference level (DRL) for one protocol.
