# Supplementary material for: Making CT Dose Monitoring Meaningful: Augmenting Dose with Imaging Quality
Source: Tomography. 2023 Apr 7;9(2):798–809. doi: 10.3390/tomography9020065 (PMC10145563; doi:10.3390/tomography9020065)
Supplement: Supplementary file 1 [file tomography-09-00065-s001.zip › tomography-2310635-supplementary.pdf]

Supplementary Materials:

Figure S1-Figure S14 show the evaluated charts in the observer study.

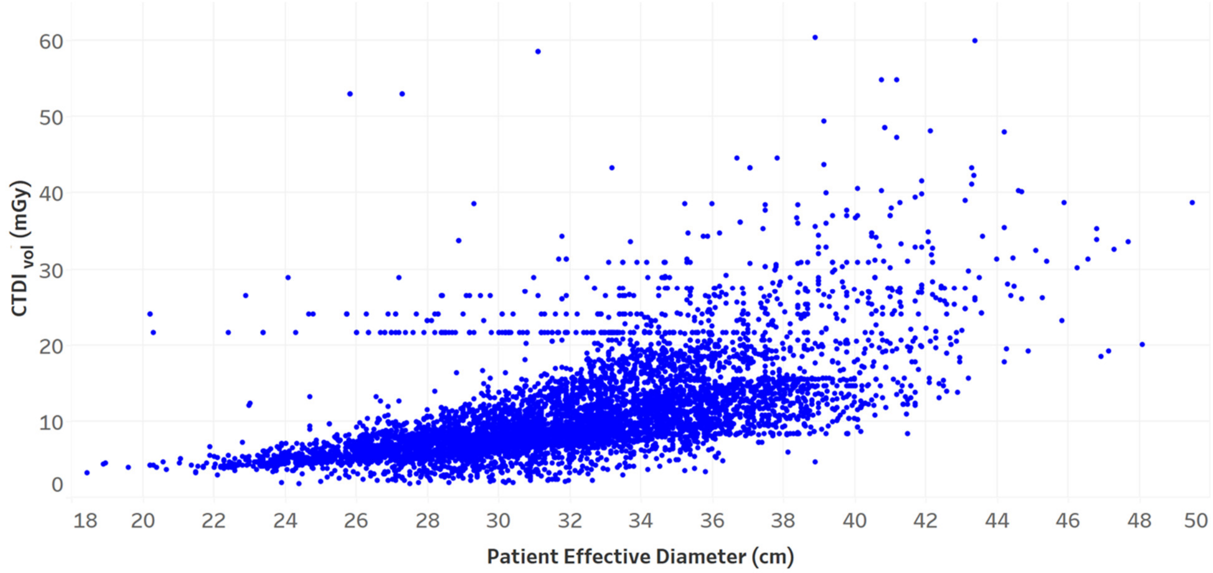

Figure S1. $CTDI_{vol}$ for patient effective diameter of all CT exams.

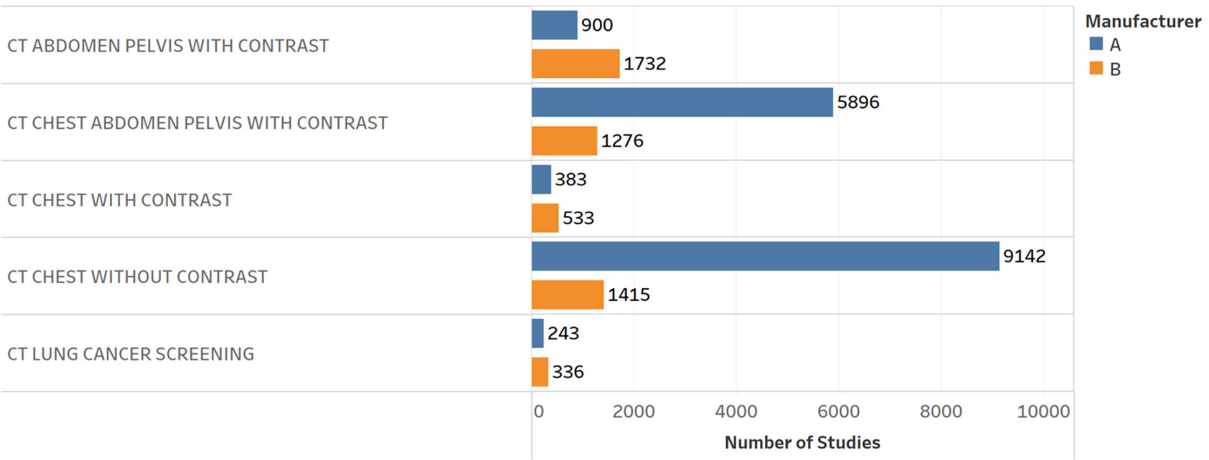

Figure S2. Distribution of CT exams by different protocols and manufacturers.

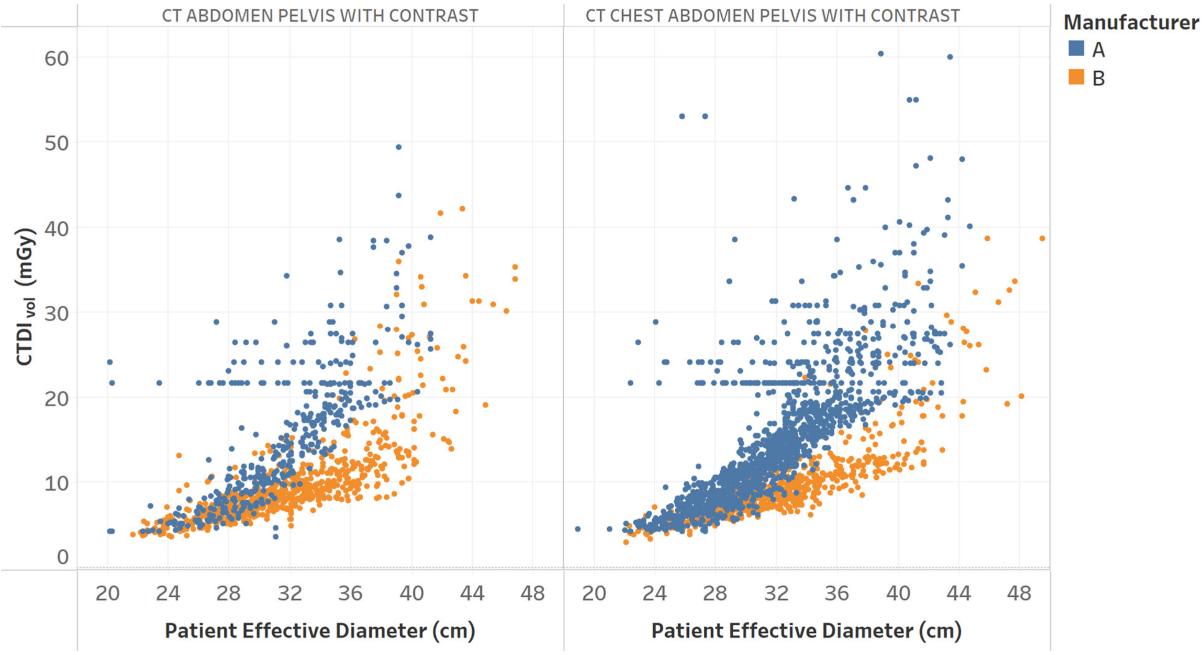

Figure S3. Distribution of the CTDIvol by different protocols, manufacturers, and patient effective diameter.

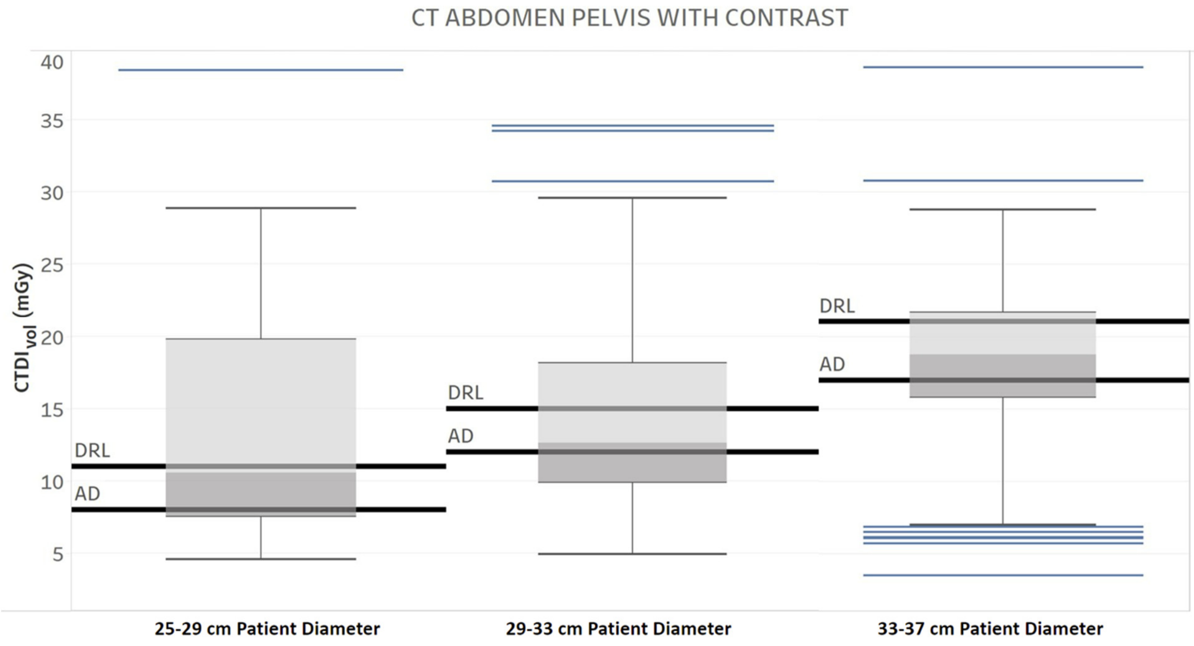

Figure S4. Diagnostic reference level (DRL) and achievable dose (AD) for three different patient diameter ranges, one protocol, and one manufacturer.

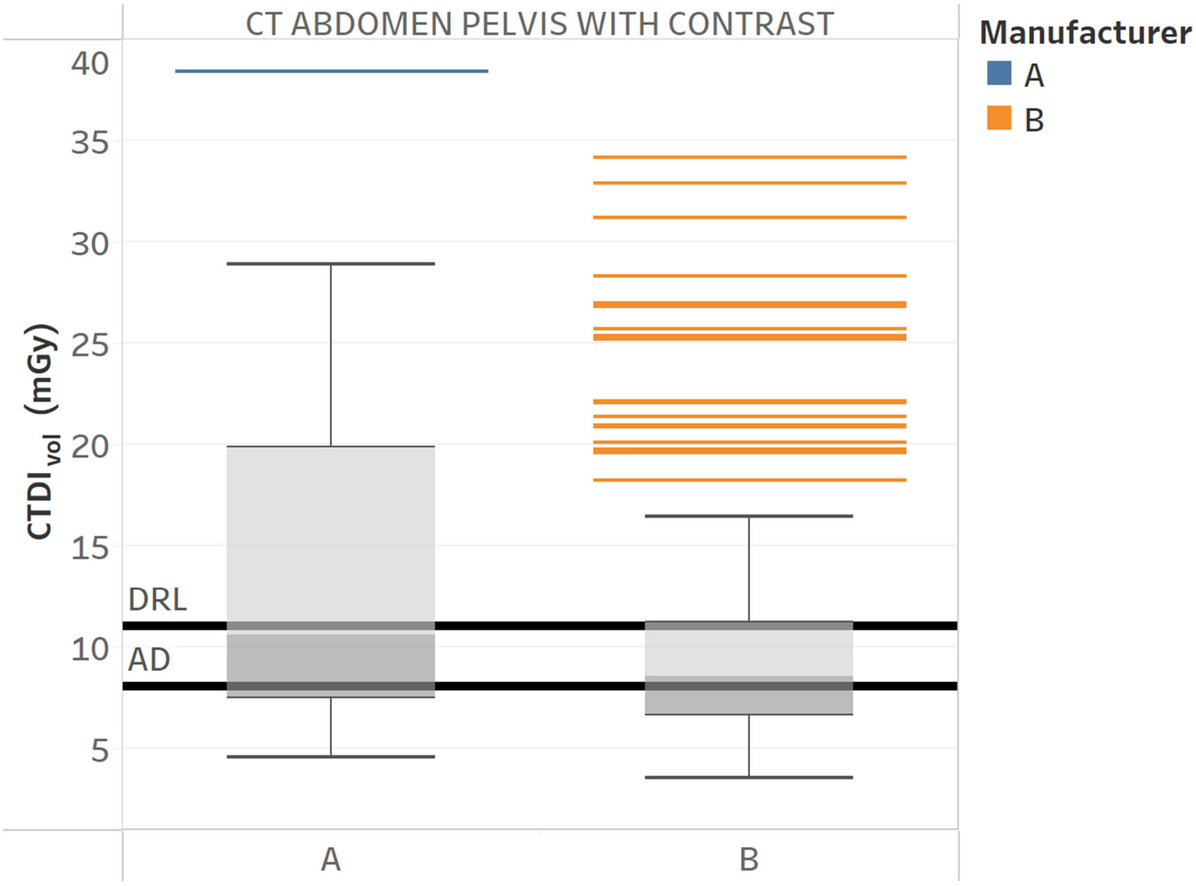

Figure S5. Diagnostic reference level (DRL) and achievable dose (AD) for one protocol and two manufacturers.

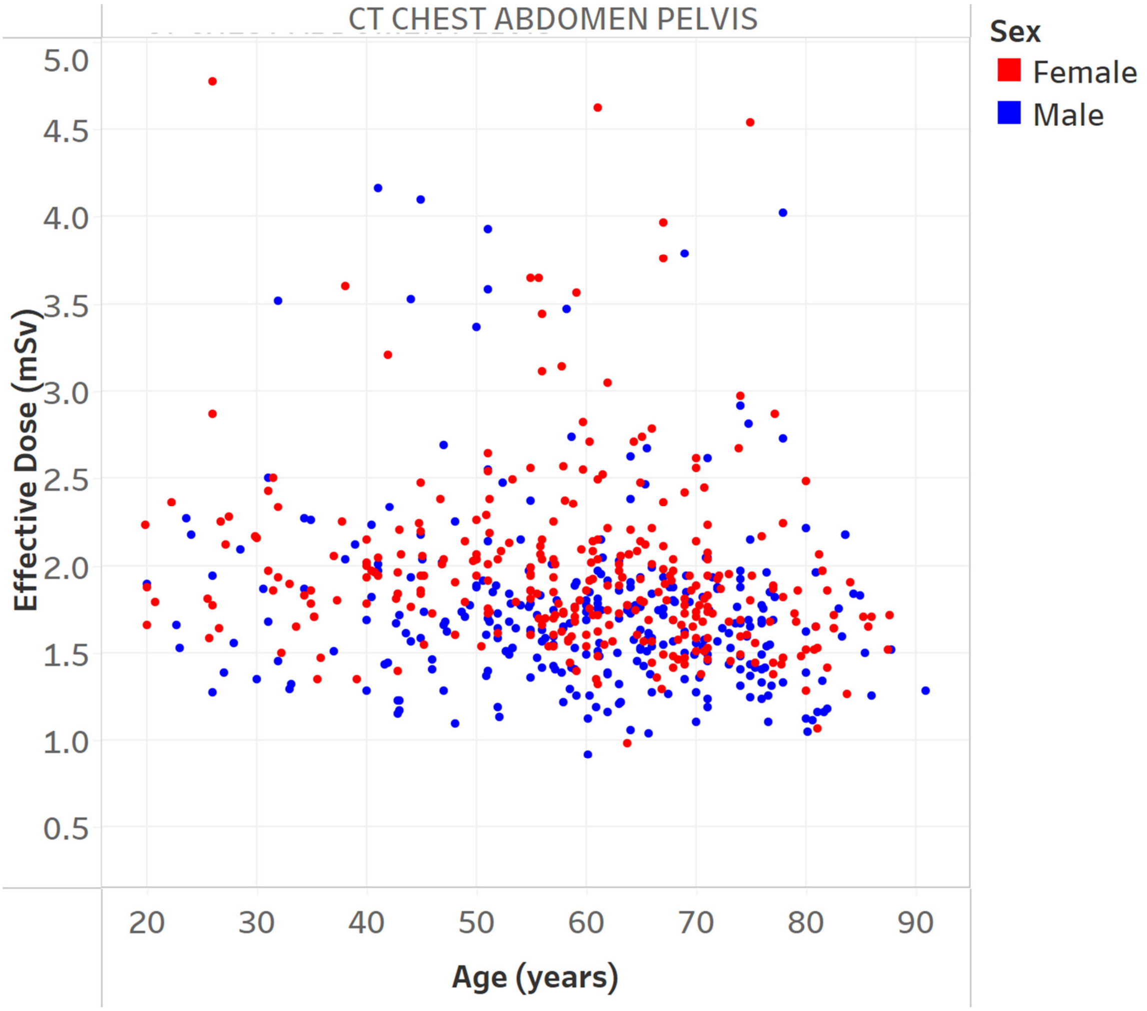

Figure S6. Effective Dose vs age for a specific protocol.

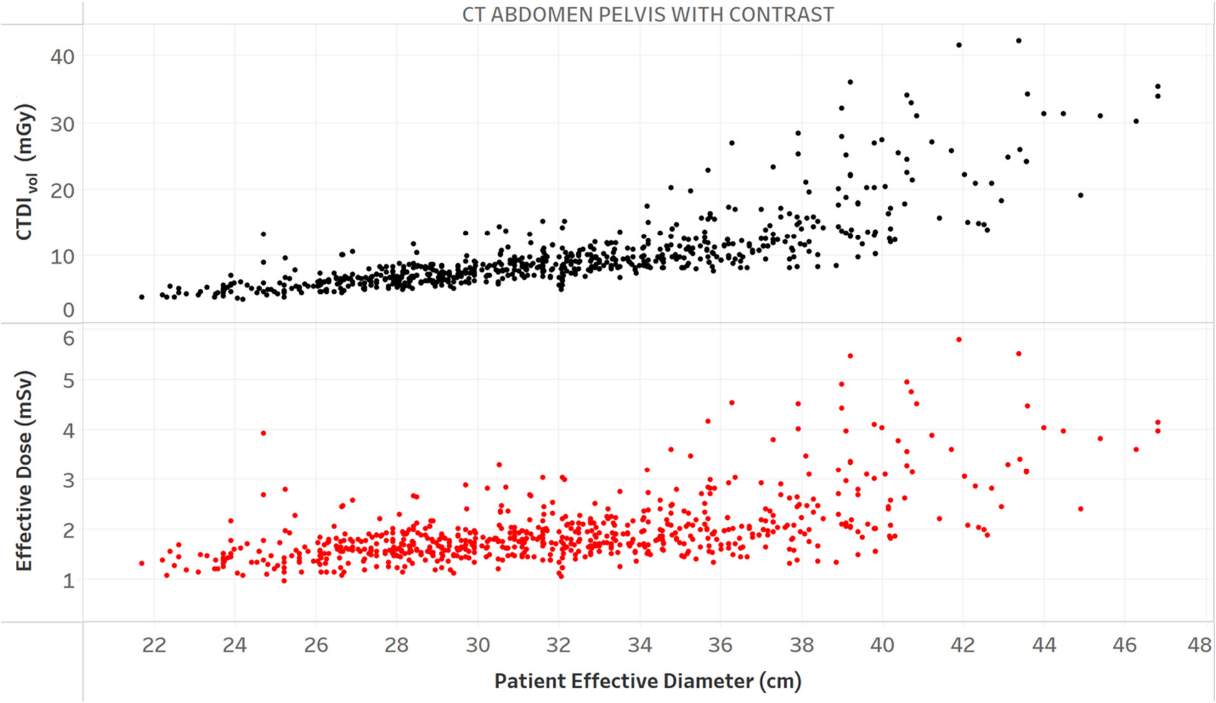

Figure S7. Distribution of the CTDIvol and effective dose vs patient diameter for one protocol and one manufacturer.

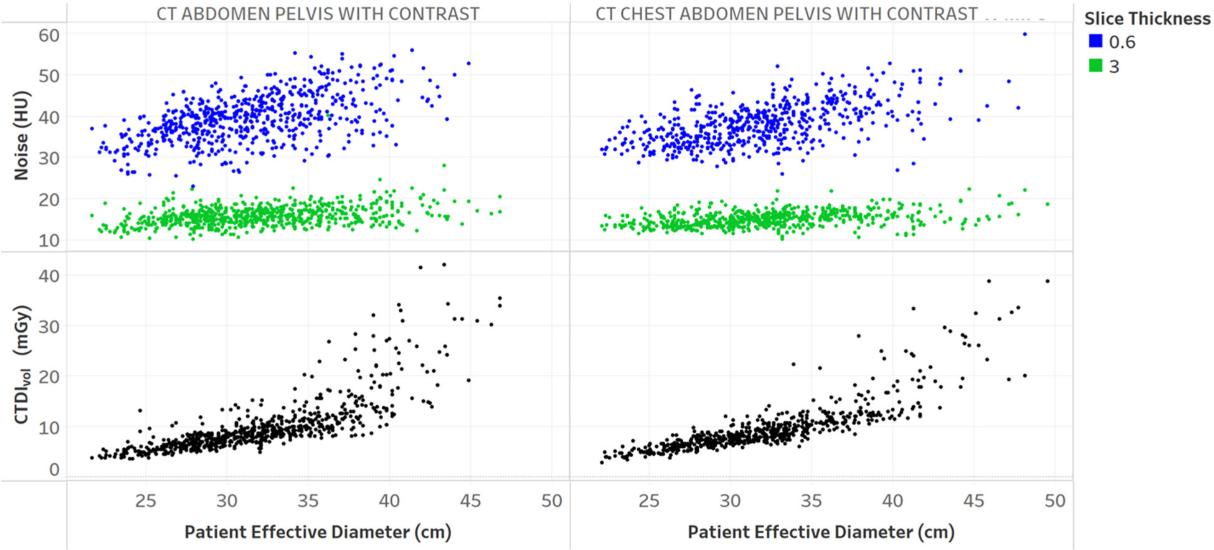

Figure S8. Distribution of noise and CTDIvol vs patient diameter for one manufacturer across two different protocols. Images were reconstructed with two different slice thicknesses.

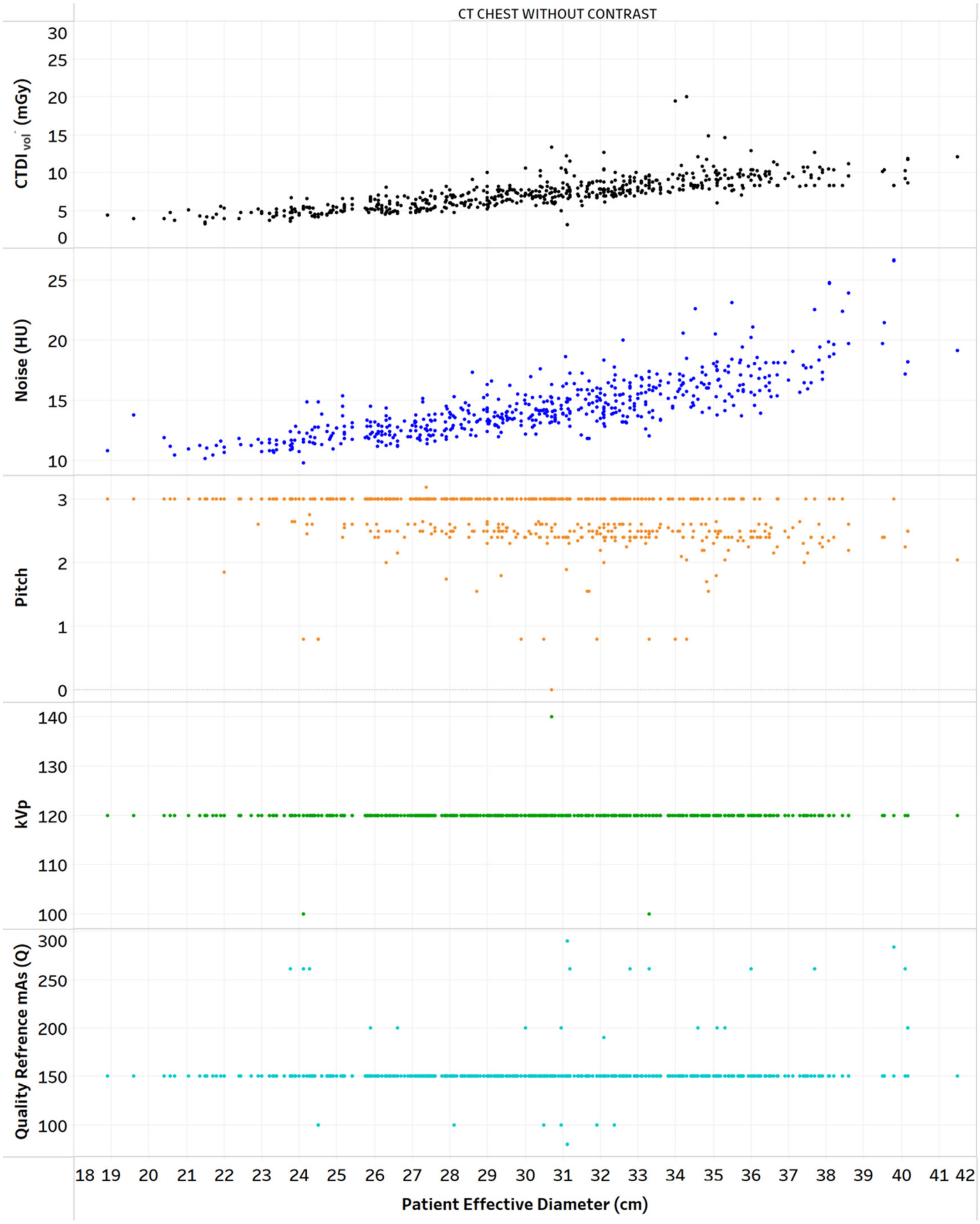

Figure S9. Distribution of $CTDI_{vol}$, noise, and scanning parameters vs patient diameter for one protocol.

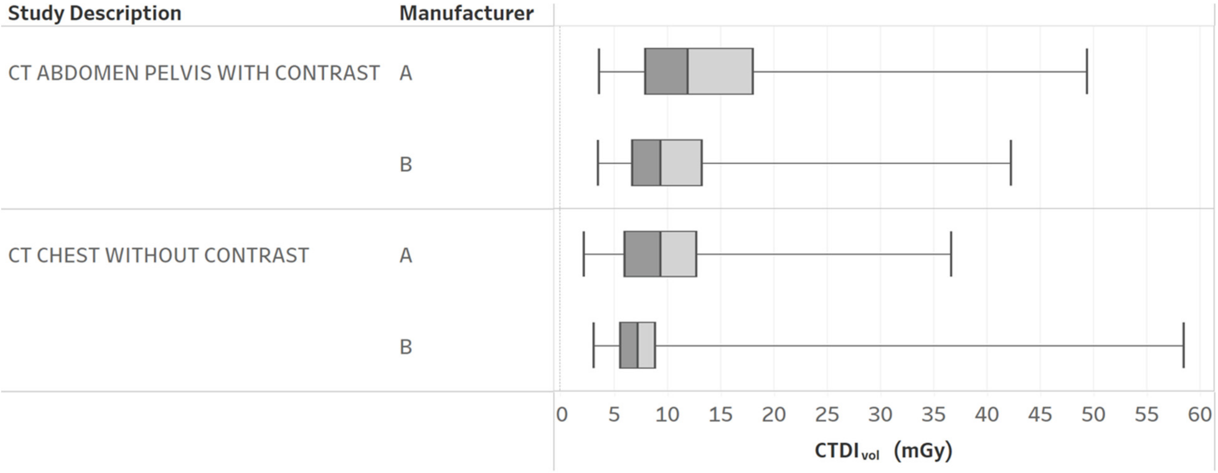

Figure S10. $CTDI_{vol}$ boxplots by different protocols and manufacturers.

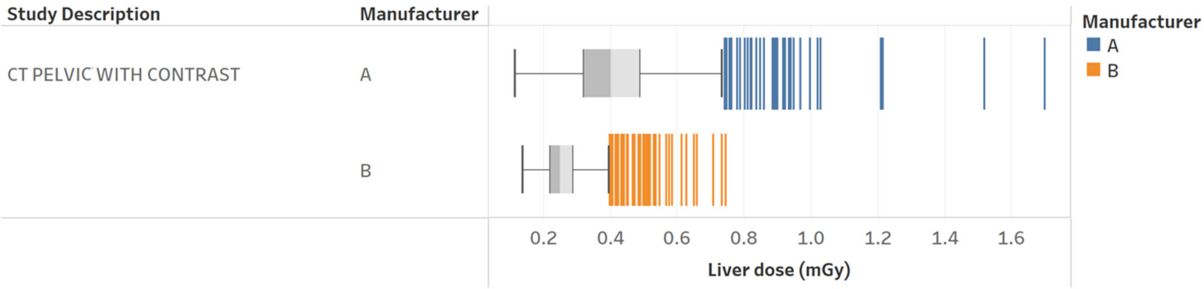

Figure S11a. Radiation dose to the liver for pelvic with contrast exams for two different scanners.

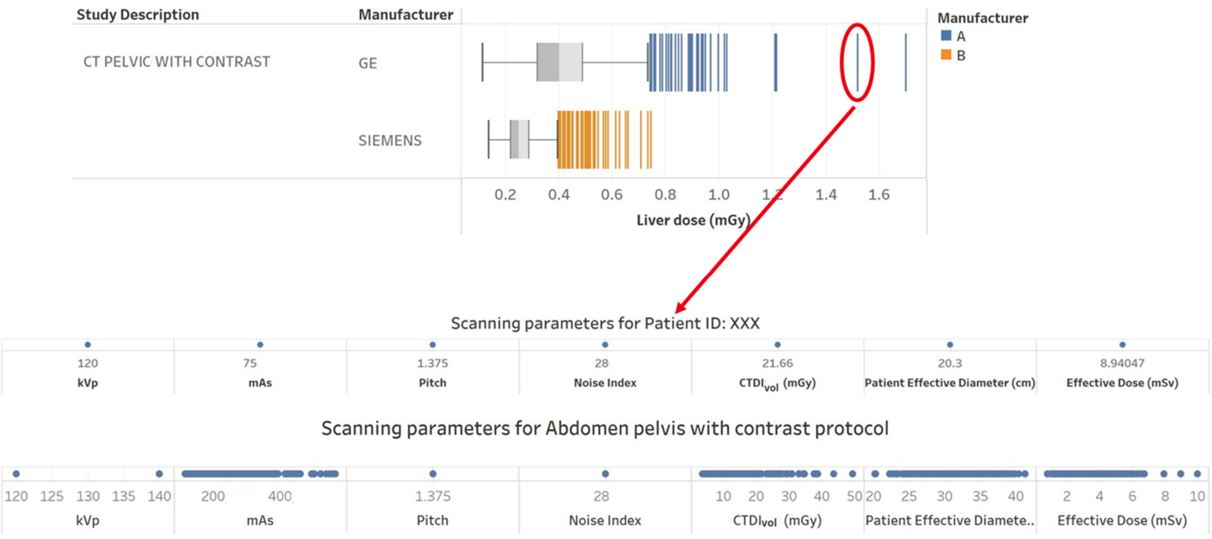

Figure S11b. Scanning parameters comparison between a specific patient (outlier) and the whole population.

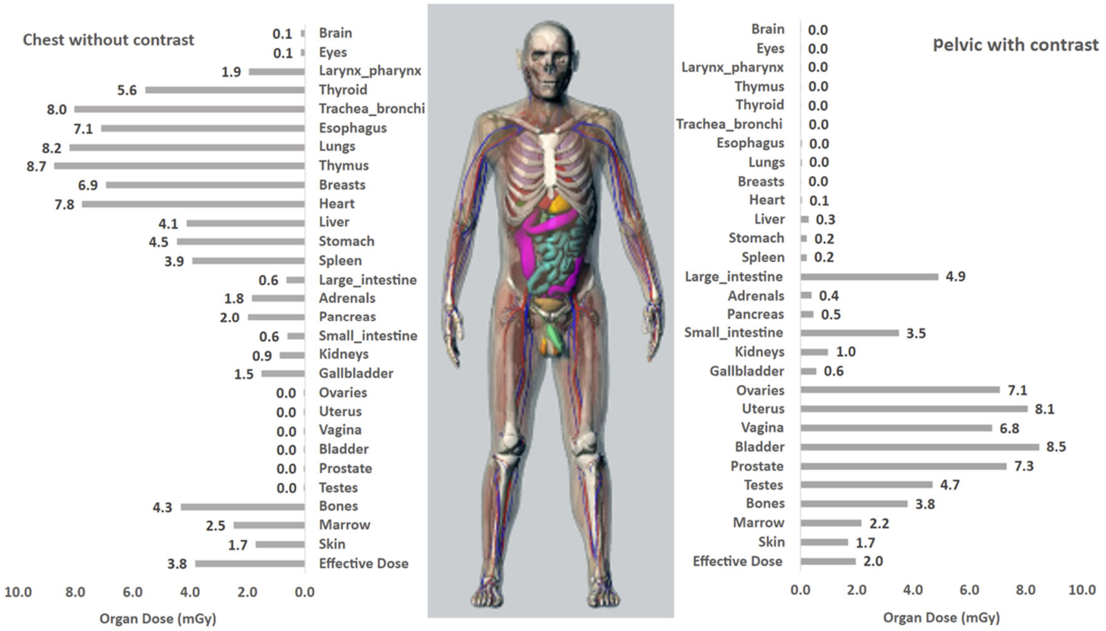

Figure S12. Median organ doses and effective dose for chest without contrast and pelvic with contrast exams.

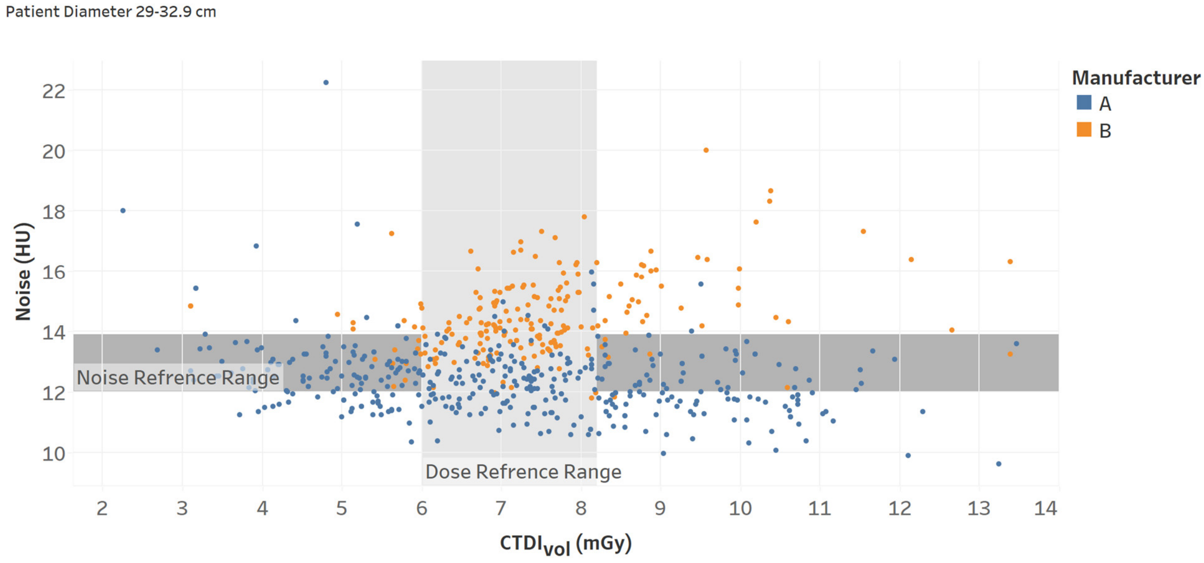

Figure S13. Noise and CTDIvol reference ranges (interquartile intervals) for a specific protocol.

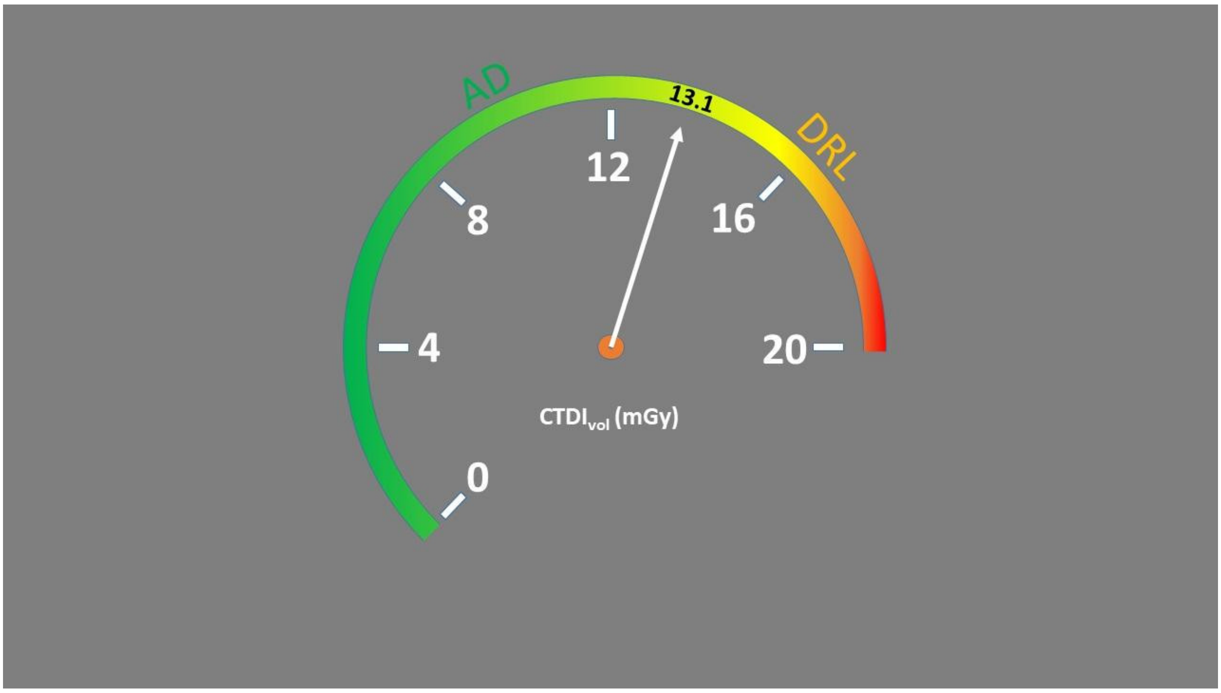

Figure S14. Comparison of institution average CTDI$_{vol}$ with achievable dose (AD) and diagnostic reference level (DRL) for one protocol.
